# Exercise‐Based Management of Diabetic Cardiomyopathy: Preclinical Mechanisms, Clinical Evidence, and Translational Strategies

**DOI:** 10.1111/1753-0407.70292

**Published:** 2026-10-30

**Authors:** Xiaoya Li, Zhiqiang Wang, Jingjing Liu

**Affiliations:** ^1^ Operating Room Department The First Affiliated Hospital of Yangtze University Jingzhou Hubei China; ^2^ Health Science Center Yangtze University Jingzhou Hubei China

**Keywords:** diabetic cardiomyopathy, exercise prescription, high‐intensity interval training, multimodal monitoring, parameter mapping

## Abstract

Diabetic cardiomyopathy is characterized by progressive metabolic dysfunction, myocardial fibrosis, impaired calcium handling, and ventricular remodeling, yet effective strategies for reversing established cardiac injury remain limited. Exercise training has emerged as a promising non‐pharmacological approach, although its clinical application is constrained by heterogeneous intervention protocols, inconsistent dose reporting, and limited evidence from well‐characterized diabetic cardiomyopathy populations. This narrative review synthesizes current preclinical and clinical evidence on exercise‐based management of diabetes‐associated myocardial dysfunction, with particular emphasis on underlying mechanisms, exercise prescription, and translational implementation. Experimental studies indicate that exercise may improve myocardial function through enhanced mitochondrial biogenesis and metabolic flexibility, attenuation of oxidative stress and inflammatory‐fibrotic signaling, restoration of calcium homeostasis, and regulation of apoptosis, pyroptosis, and microRNA‐related pathways. Clinical evidence, derived predominantly from individuals with Type 2 diabetes and subclinical or early cardiac abnormalities, suggests that combined aerobic and resistance training can improve cardiorespiratory fitness, insulin sensitivity, vascular function, and selected indices of left ventricular performance. High‐intensity interval training may provide additional benefits in appropriately selected patients, but its safety and effectiveness require individualized assessment. To improve translation, exercise intensity should be reported using physiologically comparable metrics, while treatment response should be evaluated through integrated measures of cardiac imaging, functional capacity, biomarkers, glycemic control, and adverse events. Overall, exercise represents a biologically plausible and clinically relevant adjunctive strategy for diabetic cardiomyopathy, particularly in early‐stage disease; however, adequately powered trials in clearly defined patient populations are still needed to establish optimal dose, safety, and long‐term cardiovascular benefit.

## Highlights

Exercise may improve diabetic cardiomyopathy through mitochondrial‐metabolic remodeling, restoration of redox and calcium homeostasis, and attenuation of inflammatory‐fibrotic signaling. Combined aerobic and resistance training is the practical foundation, whereas high‐intensity interval training may be considered for selected lower‐risk patients after risk stratification. Standardized dose reporting and diabetic cardiomyopathy‐specific trials are needed to define optimal efficacy, safety, and long‐term cardiovascular benefit.

## Graphical abstract

Exercise may mitigate diabetic cardiomyopathy by improving mitochondrial metabolism, redox balance, calcium handling, and inflammatory‐fibrotic remodeling. Translation into clinical practice requires phenotype‐ and risk‐adapted exercise prescriptions, standardized dose reporting, and multimodal monitoring to define safety, efficacy, and long‐term cardiovascular benefit.

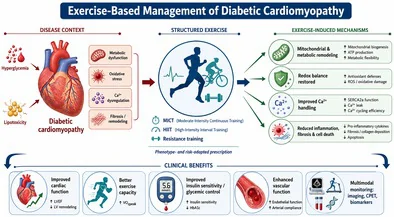

**Abbreviations**
AMPK: AMP‐activated protein kinase
BNP: B‐type natriuretic peptide
CGM: continuous glucose monitoring
CMR: cardiac magnetic resonance
CPET: cardiopulmonary exercise testing
DCM: diabetic cardiomyopathy
ECM: extracellular matrix
HF: heart failure
HFpEF: heart failure with preserved ejection fraction
HIIT: high‐intensity interval training
HRmax: maximum heart rate
HRR: heart rate reserve
LV: left ventricle/left ventricular
LVEF: left ventricular ejection fraction
LV‐HIIT: low‐volume high‐intensity interval training
MET: metabolic equivalent of task
MICT: moderate‐intensity continuous training
miRNA: microRNA
NLRP3: NOD‐like receptor family pyrin domain‐containing 3
NT‐proBNP: N‐terminal pro‐B‐type natriuretic peptide
PGC‐1α: peroxisome proliferator‐activated receptor gamma coactivator 1‐alpha
ROS: reactive oxygen species
RyR2: ryanodine receptor 2
SERCA2a: sarcoplasmic/endoplasmic reticulum calcium ATPase 2a
T1DM: Type 1 diabetes mellitus
T2DM: Type 2 diabetes mellitus
VO_2_peak: peak oxygen uptake

## 1 Introduction

Diabetic cardiomyopathy (DCM) is a distinct diabetes‐associated myocardial disorder characterized by early diastolic dysfunction, interstitial fibrosis, metabolic remodeling, and increased heart‐failure risk [1, 2, 3]. Pharmacological management of diabetes and cardiovascular risk often incompletely reverses established myocardial remodeling, supporting reproducible non‐pharmacological strategies suitable for routine prescription and monitoring [4, 5, 6, 7]. Exercise is particularly relevant because it may address systemic insulin resistance and myocardial energetic, oxidative, calcium‐handling, inflammatory‐fibrotic, and vascular abnormalities [2, 3, 4, 5, 6, 7, 8].

Preclinical and clinical evidence suggests that structured exercise, particularly moderate‐to‐vigorous aerobic plus resistance training, improves left ventricular function, cardiorespiratory fitness, and insulin sensitivity; HIIT may provide additional remodeling benefits in selected patients [4, 6, 9, 10, 11, 12, 13]. Exercise‐induced cardioprotection has also been linked to inflammasome signaling, redox‐calcium coupling, and microRNA‐mediated remodeling, while strain imaging, natriuretic peptides, and continuous glucose monitoring increasingly support individualized assessment [14, 15, 16, 17, 18, 19, 20, 21]. Translation remains limited by heterogeneous protocols, inconsistent dose reporting, and non‐harmonized endpoints linking training exposure to clinically interpretable outcomes [7, 9, 21].

Exercise studies in etiologically adjudicated DCM cohorts remain scarce. Most human evidence comes from T2DM populations with subclinical or early myocardial abnormalities and therefore applies mainly to early‐to‐intermediate phenotypes rather than advanced or definitively diagnosed DCM [1, 7, 9]. Previous reviews have usually considered mechanisms, exercise modalities, management, or imaging separately [4, 5, 7, 9, 21, 22]. This review integrates preclinical and clinical evidence around dose standardization, animal‐to‐human intensity mapping, multimodal monitoring, and priorities for DCM‐specific research.

## 2 Basic Research Evidence: Preclinical Exploration of Exercise‐Mediated Improvement in DCM

### 2.1 DCM Animal Models and Exercise Paradigms

Preclinical models of DCM are designed to capture early‐to‐intermediate phenotypes, including metabolic inflexibility, diastolic dysfunction, and interstitial fibrosis; reproducible modeling of advanced systolic failure remains difficult, which limits inference about late‐stage clinical trajectories based on these platforms alone [23]. The most commonly used models include streptozotocin (STZ)‐induced insulin‐deficient rodents and obesity/insulin‐resistance models. Their primary value lies in mechanistic interrogation and endpoint‐level evaluation under controlled conditions, while translation to long‐term clinical outcomes requires caution because experimental disease course, comorbidity structure, and background treatment context differ from those of clinical populations [23].

Treadmill‐based MICT with progressive overload predominates in preclinical DCM research; HIIT and swimming are less common, limiting modality‐ and intensity‐specific inference [24, 25, 26]. Across models, 8–12 weeks of aerobic or interval training improves cardiac performance and diastolic filling and preserves myocardial histology and ultrastructure [24, 27, 28]. These linked readouts support alignment of training exposure with functional, structural, and cellular endpoints when outcomes are prespecified and measured consistently [24, 27, 28].

Preclinical and clinical studies report exercise dose using non‐equivalent systems: animal protocols use speed, grade, and duration, whereas clinical prescriptions use METs, %VO_2_peak, and HRR. Iso‐metabolic calibration—linking animal speed to oxygen consumption and human intensity to HRR—would compare physiological rather than nominal workloads, improving cross‐study comparability and dose–response inference [9]. Table 1 summarizes representative models, prescriptions, and endpoints to align “what to train” with “what to measure.”

**TABLE 1:** Summary of animal models and exercise paradigms.

Model/species	Diabetic phenotype	Exercise modality	Typical prescription	Key mechanistic domains^a^	Main structural/functional outcomes	References
STZ rats (T1DM)	Advanced DCM; hypertrophy and fibrosis	MICT (treadmill)	5 days/week; 30–60 min; 8–10 weeks	Mitochondrial biogenesis; metabolic remodeling	↓Myocardial injury; ↓fibrosis; ↑LV function	[29]
STZ rats (T1DM)	DCM; remodeling	Swimming	5–7 days/week; 30–60 min; several weeks	Oxidative stress; ECM remodeling	↓LV remodeling; ↑diastolic indices	[30]
STZ rats (T1DM)	Severe DCM	HIIT (treadmill)	Interval protocol; ~8 weeks	Nitro‐redox balance; fibrosis	↓Fibrosis and hypertrophy; limited redox recovery	[31]
db/db mice (T2DM)	Obesity; insulin resistance; LV dysfunction	MICT (treadmill)	30–60 min; several weeks	Mitochondrial function; gap junctions	↑Systolic and diastolic function	[32]
ZDF rats (T2DM)	Obesity‐related DCM	MICT (treadmill)	Mid‐long term	Insulin signaling	↑Contractility; improved signaling	[33]
GK rats (T2DM)	Endothelial dysfunction	MICT (treadmill)	Several weeks	Endothelial NO signaling	↑Microvascular and LV function	[34]
T2DM rats	Myocardial fibrosis	MICT (treadmill)	Long‐term; moderate	Oxidative stress; TGF‐β/Smad	↓Fibrosis; ↑LV compliance	[35]
HFD mice (T2DM)	DCM phenotype	MICT (treadmill)	~16 weeks	Inflammation/pyroptosis	↓Fibrosis; ↑LV function	[36]
T2DM rats	Fibrotic DCM	Swimming	5 days/week; 60 min; 8 weeks	miRNA‐mediated metabolic remodeling	↓Fibrosis; ↑EF/FS	[37]
STZ rats (T1DM)	DCM	HIIT (treadmill)	5 days/week; 5–8 weeks	miRNA‐dependent apoptosis	↑EF/FS; ↓apoptosis	[38]
HFHF diet rats (T2DM)	Obesity‐related DCM	HIIT	Several weeks	miRNA‐mediated remodeling	↑Cardiac function; ↓remodeling	[39]

*Note:* Data are derived from representative preclinical studies in rodent models of diabetic cardiomyopathy. Unless otherwise specified, exercise interventions consisted of supervised treadmill running or swimming. Exercise intensity was most often graded according to running speed or estimated %VO_2_max and implemented as moderate‐intensity continuous training (MICT), HIIT, or comparisons between these modalities. In the included reports, deaths or clearly defined serious adverse events directly attributable to the exercise intervention were not reported.

Abbreviations: DCM, diabetic cardiomyopathy; ECM, extracellular matrix; EF, ejection fraction; FS, fractional shortening; GK, Goto‐Kakizaki; HFD, high‐fat diet; HFHF, high‐fat high‐fructose diet; HIIT, high‐intensity interval training; LV, left ventricle; LV‐HIIT, low‐volume high‐intensity interval training; MICT, moderate‐intensity continuous training; miRNA, microRNA.; STZ, streptozotocin; T1DM, Type 1 diabetes mellitus; T2DM, Type 2 diabetes mellitus; ZDF, Zucker diabetic fatty.

^a^ Key mechanistic domains correspond to Sections 3.1, 3.5 (mitochondrial‐metabolic remodeling, redox balance, Ca^2+^ handling, fibrosis/cell death, and miRNA/epigenetic regulation).

### 2.2 Translational Limitations of Preclinical Research

Cross‐species dose translation constrains the clinical applicability of preclinical exercise research because “exposure” is quantified with non‐equivalent constructs in animal and human studies. Animal experiments typically operationalize exercise dose using treadmill speed, grade, and duration, whereas clinical prescriptions are defined using physiological indices such as metabolic equivalents (METs), %VO_2_peak, or heart‐rate reserve (HRR). Given that an identical nominal speed can correspond to different relative metabolic loads across species—and can be further modified by strain, age, and disease severity—speed‐based extrapolation does not preserve exposure equivalence. This mismatch provides a logical basis for why dose–response relationships may be misestimated and why findings may be difficult to reproduce across laboratories and translational steps [9, 23].

Improving comparability requires dose reporting that is explicitly anchored to metabolic demand rather than to absolute external workload. An iso‐metabolic load framework addresses this requirement by expressing intensity in relative physiological terms that can be mapped between preclinical and clinical contexts. In animal protocols, incorporating indirect calorimetry (VO_2_/RER) during familiarization or representative sessions enables calibration of the speed‐VO_2_ relationship, allowing intensity to be reported as %VO_2_max or VO_2_ reserve equivalents instead of treadmill speed alone. In clinical studies, defining intensity zones using HRR or %VO_2_peak and reporting weekly effective minutes, attendance, and time‐in‐zone provides an exposure description that can be aligned with calibrated preclinical intensities and compared across cohorts and interventions without assuming that external workload is equivalent to physiological stress [9].

Evidence synthesis is additionally limited by heterogeneity arising from incomplete reporting and variable protocol progression. Preclinical studies often incompletely document adherence, sex and age distribution, comorbidity burden, and background treatments, and laboratories differ in training progression parameters, including grade settings, intensity increments, and intervention duration. These deviations introduce between‐study variability that weakens meta‐analytic interpretability and complicates causal attribution because observed effects cannot be confidently separated from differences in exposure implementation and background conditions [23]. Sex‐related differences in cardiac metabolism and Ca^2+^ handling have been described; prospective sex stratification and interaction testing are therefore necessary for conclusions intended to inform individualized exercise prescriptions, because the absence of these design elements leaves uncertainty regarding effect modification by sex [23].

A further limitation is the translation of mechanistic readouts to outcomes used for clinical decision‐making. Animal studies frequently report molecular/cellular and histological improvements, yet inference about reverse remodeling, functional capacity, and prognosis requires DCM‐focused, adequately powered human trials that apply harmonized endpoints and reporting standards. Without such alignment, mechanistic improvements remain insufficient for judging clinical effectiveness or for estimating the magnitude of benefit relevant to patients [7, 9]. A reporting template that links “animal training paradigms → clinically interpretable intensity zones” can therefore function as a dose‐reporting framework rather than a prescription (Table 2). The template emphasizes standardized dose metrics and adherence reporting and specifies cross‐species outcome anchors—cardiac imaging (e.g., strain and T1 mapping when feasible), functional capacity (cardiopulmonary exercise testing (CPET)/VO_2_peak), and circulating biomarkers (BNP/NT‐proBNP)—while positioning multi‐omics readouts as mechanistic secondary endpoints to support, rather than replace, clinically interpretable outcomes [1, 7, 9, 45].

**TABLE 2:** Template for mapping exercise intensity from animals to clinical practice.

Template level	Animal‐side paradigm	Inferred relative load (animals)	Corresponding clinical intensity	Minimal adherence/dose recording	References
Low‐intensity adaptation/severe stage	8–10 m/min, 0°–5°, 20–30 min, 3–5 days/week	~30%–40% VO_2_max	~30%–40% VO_2_R (≈30%–40% HRR), ~2–3 METs	Weekly device‐minutes; attendance ≥ 70%; time‐in‐zone documented	[40, 41]
MICT (moderate continuous)	~12 m/min, 0°, 30–60 min, ~5 days/week (8–12 weeks)	~50%–60% VO_2_max	~40%–60% VO_2_R (≈40%–60% HRR), ~3–6 METs	Target ≥ 150 min/week in 40%–60% HRR; attendance ≥ 75%	[9, 40, 41, 42]
Near‐threshold (moderate–high)	16–18 m/min, 5°–10°, 40–50 min, 3–5 days/week	~60%–75% VO_2_max	~60%–75% VO_2_R (≈60%–75% HRR), ~5–8 METs	Prefer CPET‐calibrated HR zones; report minutes in‐zone and adverse events	[9, 40, 41, 42]
HIIT (standard)	4 × 4 min (work) with recovery; 2–3 sessions/week (example)	Work: ~80%–90% VO_2_max	Work: ~70%–90% VO_2_R (≈70%–90% HRR)	Report work‐interval minutes; time > 70% HRR; RPE 15–17; attendance ≥ 80%	[9, 41, 42]
LV‐HIIT (low‐volume)	Shorter total work duration per session (e.g., ≤ 10 to 15 min work)	Work: ~80%–90% VO_2_max (session load lower)	Work: ~70%–90% VO_2_R (≈70%–90% HRR)	Report total high‐intensity work minutes and weekly session count; monitor adverse events	[42, 43, 44]

*Note:* This table is intended as a dose‐reporting and intensity‐mapping template to improve comparability between preclinical and clinical studies rather than a patient‐specific exercise prescription. Clinical implementation should be individualized based on CPET, comorbidities, and safety assessment.

Abbreviations: CPET, cardiopulmonary exercise testing; HIIT, high‐intensity interval training; HRR, heart rate reserve; LV‐HIIT, low‐volume high‐intensity interval training; MET, metabolic equivalent of task; MICT, moderate‐intensity continuous training; RPE, rating of perceived exertion; VO_2_peak, peak oxygen uptake; VO_2_R, oxygen uptake reserve.

## 3 Core Mechanisms by Which Exercise Ameliorates DCM

### 3.1 Regulation of Mitochondrial Function and Metabolic Remodeling

DCM involves impaired metabolic flexibility, reflected by reduced glucose utilization, a relative shift toward fatty acid oxidation (FAO), and inefficient mitochondrial coupling. These disturbances are linked to increased reactive oxygen species (ROS) production and progressive mechanical dysfunction, with diastolic impairment frequently appearing early in the disease course [2, 3, 4, 5, 6, 7, 8]. Exercise training is posited to modify this trajectory by acting on the coupled determinants of myocardial energetics—mitochondrial performance, substrate handling, and contraction‐relaxation efficiency—with mechanistic support derived primarily from experimental and translational studies [2, 3, 4, 5, 6, 7, 8, 46, 47, 48].

Evidence connecting exercise to mitochondrial remodeling centers on the PGC‐1α/nuclear respiratory factor 1 (NRF1)/TFAM axis, a regulatory network implicated in mitochondrial biogenesis and oxidative phosphorylation capacity [4, 5, 6, 7, 47, 48]. In diabetic models, exercise‐associated upregulation of this pathway coincides with improved markers of myocardial energetics and with functional correlates that include improved diastolic filling and global cardiac performance [4, 5, 6, 7]. The mechanistic link is logically coherent because relaxation is ATP‐dependent and mitochondrial coupling efficiency constrains ATP availability; however, the extent to which signaling activation precedes functional recovery, rather than tracking with it, is expected to vary with disease severity and training dose [2, 3, 4, 5, 6, 7, 8].

A parallel line of mechanistic evidence implicates exercise‐induced activation of the liver kinase B1 (LKB1)‐AMP‐activated protein kinase (AMPK) pathway as an energy‐sensing regulator of substrate utilization (Figure 1) [5, 6, 7, 46]. AMPK facilitates glucose transporter type 4 (GLUT4) translocation and glucose uptake and, through phosphorylation‐mediated inhibition of acetyl‐CoA carboxylase (ACC), lowers malonyl‐CoA and reduces inhibition of carnitine palmitoyltransferase 1 (CPT1), enabling adjustment of mitochondrial FAO flux away from a maladaptive “excessive FAO” state [5, 6, 7, 46]. This substrate rebalancing provides a mechanistic rationale for limiting accumulation of lipotoxic intermediates and lowering mitochondrial ROS burden, which aligns with reported improvements in relaxation and contractile efficiency in diabetic settings [3, 5, 6, 7, 8].

**FIGURE 1:**
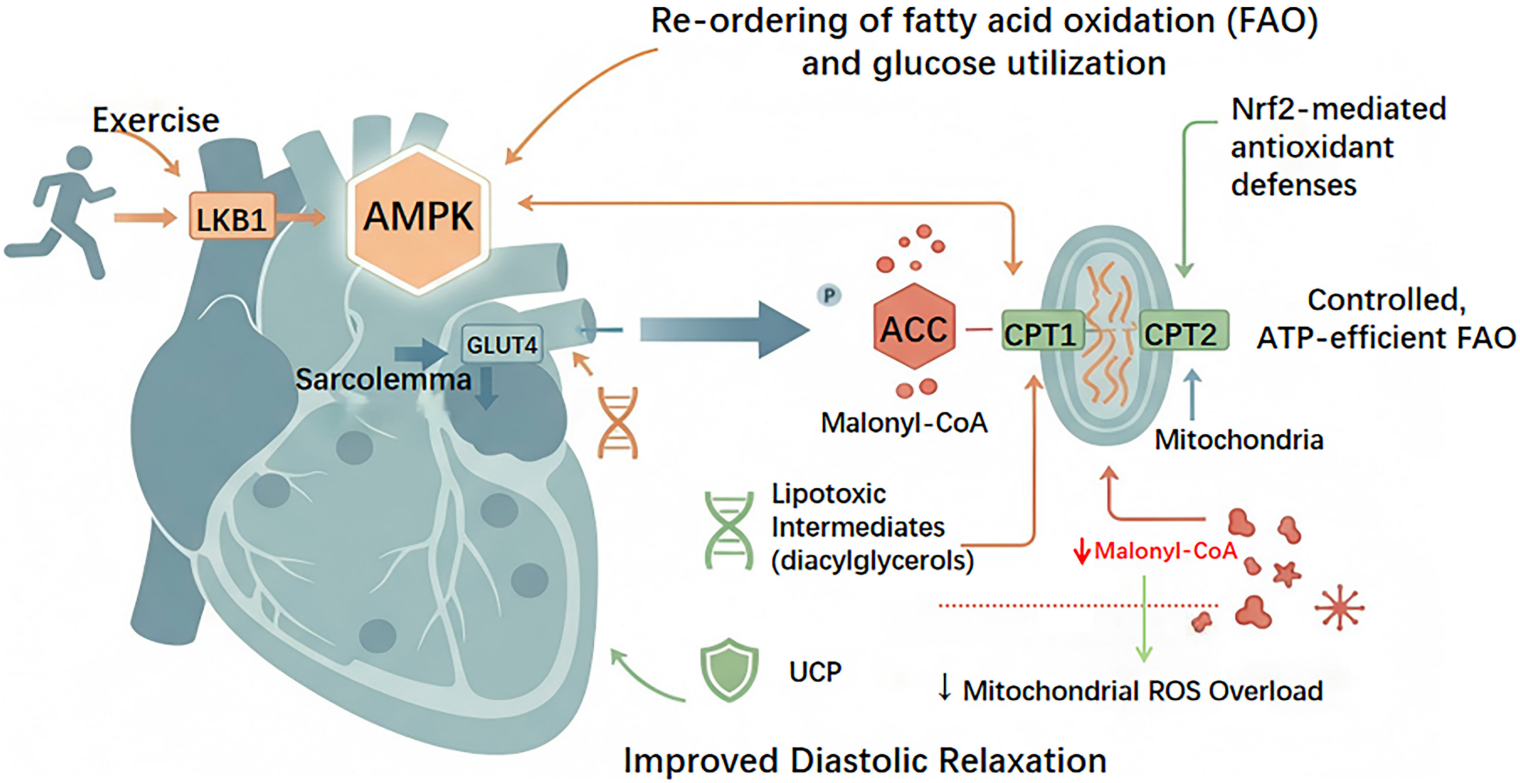
Exercise‐driven substrate reordering via the LKB1‐AMPK‐ACC‐CPT1 axis attenuates mitochondrial oxidative stress and improves diastolic relaxation. Exercise reprograms myocardial substrate utilization in DCM through the LKB1‐AMPK‐ACC‐CPT1 axis by enhancing glucose uptake, reducing excessive fatty acid oxidation, alleviating mitochondrial oxidative stress, and improving diastolic relaxation. FAO, fatty acid oxidation; LKB1, liver kinase B1; AMPK, AMP‐activated protein kinase; GLUT4, glucose transporter type 4; ACC, acetyl‐CoA carboxylase; CoA, coenzyme A; CPT1, carnitine palmitoyltransferase 1; CPT2, carnitine palmitoyltransferase 2; ATP, adenosine triphosphate; Nrf2, nuclear factor erythroid 2‐related factor 2; UCP, uncoupling protein; ROS, reactive oxygen species.

Within this framework, exercise‐associated PGC‐1α‐linked mitochondrial adaptation and AMPK‐mediated substrate reprogramming represent mechanistically convergent routes to metabolic correction in diabetes‐associated myocardial dysfunction phenotypes, mechanisms that are considered relevant to the pathophysiology of DCM [3, 4, 5, 6, 7, 8, 46, 48]. The same reasoning supports parameterizing exercise intensity and dose according to insulin resistance and glucolipotoxic burden, because these phenotypic constraints are expected to alter the relative contributions of substrate selection, mitochondrial coupling, and oxidative stress to contractile dysfunction [4, 5, 6, 7].

### 3.2 Oxidative Stress and Antioxidant System Balance

In diabetes‐associated myocardial dysfunction (predominantly studied in Type 2 diabetes mellitus (T2DM) cohorts and experimental models relevant to DCM), persistent hyperglycemia and lipotoxicity are linked to excessive ROS generation, with NADPH oxidase (NOX) isoforms and mitochondria representing major sources. ROS accumulation disrupts redox homeostasis, promotes endothelial nitric oxide synthase (eNOS) uncoupling, reduces nitric oxide (NO) bioavailability, and is associated with the amplification of inflammatory‐fibrotic remodeling [5, 7, 49, 50, 51, 52]. Structured exercise has been proposed to modulate this process through concurrent effects on ROS production and antioxidant capacity.

Existing evidence indicates that training attenuates NOX activation and superoxide generation, reducing upstream oxidative load, while also enhancing endogenous antioxidant defenses—including superoxide dismutase (SOD), glutathione peroxidase (GSH‐Px), and catalase (CAT)—with concomitant reductions in lipid peroxidation and protein nitration and a shift toward redox re‐equilibration (Figure 2) [5, 53, 54, 55].

**FIGURE 2:**
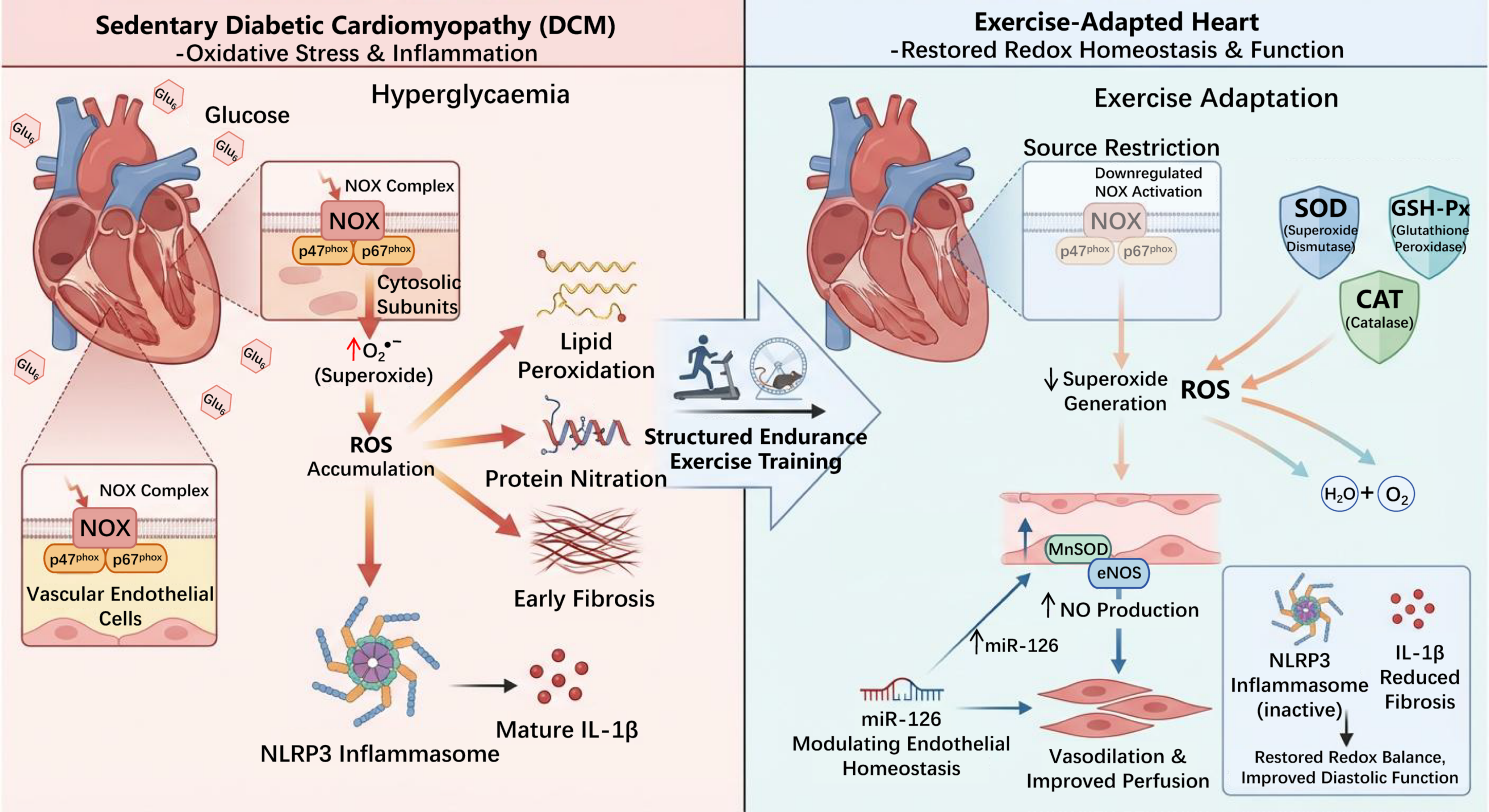
Proposed mechanisms by which endurance exercise attenuates oxidative stress and inflammatory signaling in diabetic cardiomyopathy. Exercise attenuates oxidative stress in DCM by suppressing ROS production from NOX and mitochondria and by enhancing endogenous antioxidant defenses, thereby improving endothelial function and limiting inflammatory and fibrotic remodeling. CAT, catalase; DCM, diabetic cardiomyopathy; eNOS, endothelial nitric oxide synthase; GSH‐Px, glutathione peroxidase; H_2_O, water; IL‐1β, interleukin‐1 beta; miR‐126, microRNA‐126; MnSOD, manganese superoxide dismutase; NLRP3, NOD‐like receptor family pyrin domain‐containing 3; NO, nitric oxide; NOX, NADPH oxidase; O_2_, oxygen; O_2_•−, superoxide anion; p47phox, 47‐kDa phagocyte oxidase subunit; p67phox, 67‐kDa phagocyte oxidase subunit; ROS, reactive oxygen species; SOD, superoxide dismutase.

Exercise improves endothelial function and perfusion‐function coupling even without weight loss or glycemic normalization, supporting a training‐specific vascular effect [56]. Restored redox balance may reduce ROS‐driven inflammation and fibrosis in diabetes‐related myocardial remodeling [7, 15, 49]. Responses depend on dose and timing: higher‐intensity training may reduce hypertrophy and fibrosis, whereas excessive early loading may not normalize nitroso‐redox coupling, requiring calibrated prescriptions and direct validation in adjudicated DCM cohorts [31]. The “source restriction plus sink augmentation” model summarizes how exercise may restore redox homeostasis and protect cardiac function [5, 7, 54].

### 3.3 Ca^2+^ Homeostasis and Restoration of Myocardial Contractile Function

In DCM, excitation‐contraction coupling impairment can be traced to abnormalities in intracellular Ca^2+^ cycling and disruption of microdomain coupling. Evidence indicates that reduced activity of sarcoplasmic/endoplasmic reticulum Ca^2+^‐ATPase 2a (SERCA2a), increased diastolic Ca^2+^ leak via ryanodine receptor 2 (RyR2), and loss of transverse‐tubule (T‐tubule) integrity jointly compromise the spatiotemporal coordination of Ca^2+^ release and reuptake [7, 57, 58, 59, 60]. At the functional level, these changes correlate with early diastolic relaxation impairment and, with progression of remodeling, are linked to secondary systolic dysfunction [7, 57, 60].

Exercise training is reported to improve Ca^2+^ handling by shifting both Ca^2+^ reuptake capacity and release synchronization toward a more coordinated state (Figure 3). Studies cited in this section support an increase in sarcoplasmic reticulum (SR) Ca^2+^ reuptake associated with enhanced SERCA2a function and more stable regulation, which is consistent with faster diastolic Ca^2+^ clearance and improved relaxation [24]. Other evidence links exercise to preservation or restoration of T‐tubule organization and improved synchrony of Ca^2+^ release, changes that align with improved efficiency of systolic activation and cardiomyocyte shortening [24, 61]. Oxidative stress modulation provides a mechanistic bridge between metabolic stress and Ca^2+^ leak, because oxidative stress promotes maladaptive activation of calcium/calmodulin‐dependent protein kinase II (CaMKII) and destabilization of RyR2. Exercise‐associated reductions in oxidative stress are therefore consistent with attenuation of the Ca^2+^ “leak‐overload” process and with a reduction in the energetic burden required to maintain Ca^2+^ cycling under pathological conditions [24, 31, 62].

**FIGURE 3:**
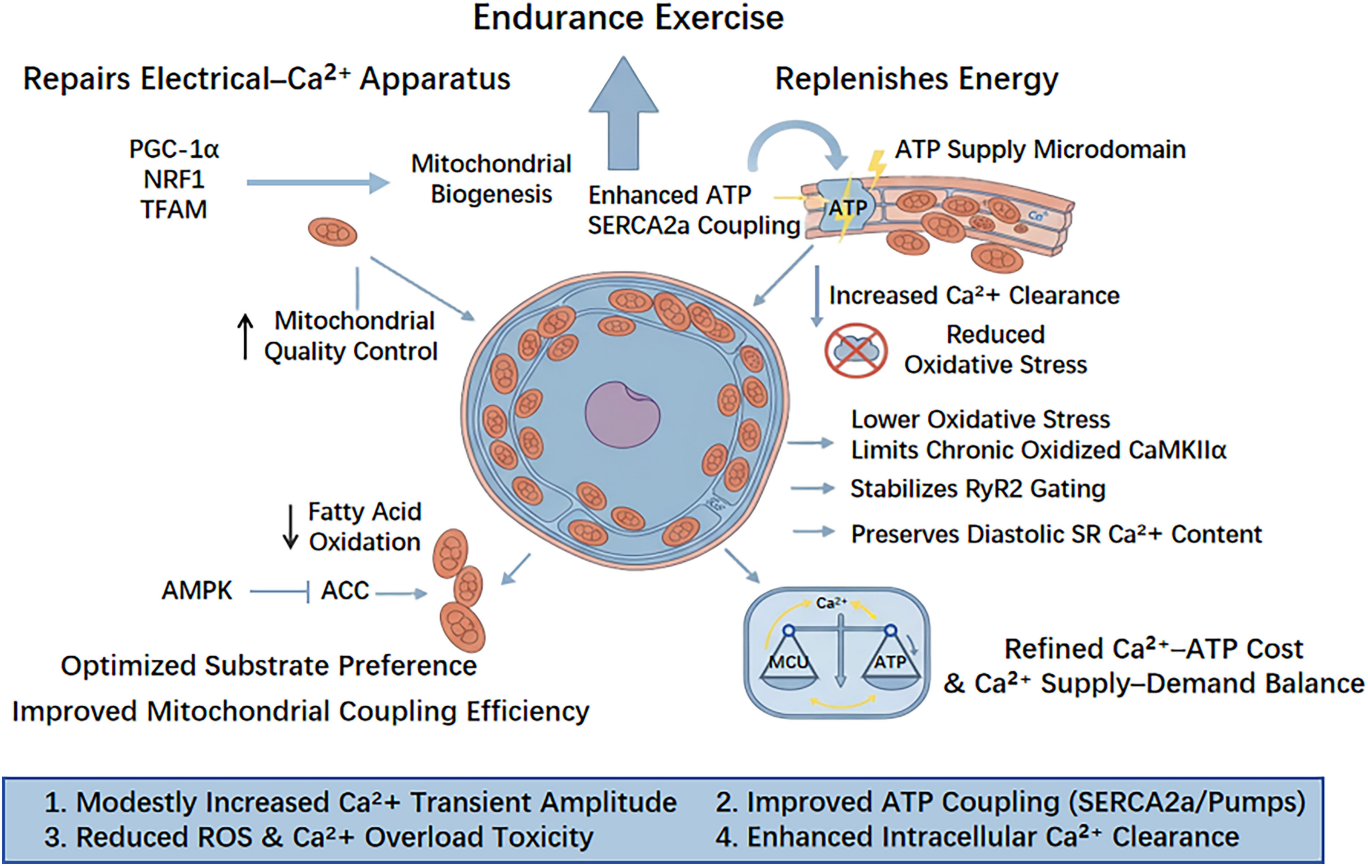
Endurance exercise rebuilds cardiomyocyte Ca^2+^‐ATP coupling by repairing the electrical‐Ca^2+^ handling apparatus and replenishing energy. Exercise restores cardiomyocyte Ca^2+^‐ATP coupling in DCM by improving SERCA2a‐mediated Ca^2+^ reuptake, reducing RyR2‐associated Ca^2+^ leak, preserving T‐tubule integrity, and supporting more coordinated excitation‐contraction coupling. ACC, acetyl‐CoA carboxylase; AMPK, AMP‐activated protein kinase; ATP, adenosine triphosphate; Ca^2+^, calcium; CaMKIIα, calcium/calmodulin‐dependent protein kinase II alpha; MCU, mitochondrial calcium uniporter; NRF1, nuclear respiratory factor 1; PGC‐1α, peroxisome proliferator‐activated receptor gamma coactivator 1‐alpha; ROS, reactive oxygen species; RyR2, ryanodine receptor 2; SERCA2a, sarcoplasmic/endoplasmic reticulum Ca^2+^‐ATPase 2a; SR, sarcoplasmic reticulum; TFAM, mitochondrial transcription factor A.

The energy‐Ca^2+^ interface constrains the magnitude and durability of these adaptations (Figure 4), since SERCA2a and sarcolemmal ion pumps operate with direct ATP dependence. Exercise‐induced mitochondrial remodeling is linked to improved microdomain ATP availability for SR and membrane transport systems and to improved metabolic efficiency; both changes plausibly reduce oxidative burden and thereby limit reinforcement of Ca^2+^ dysregulation [7, 58, 59].

**FIGURE 4:**
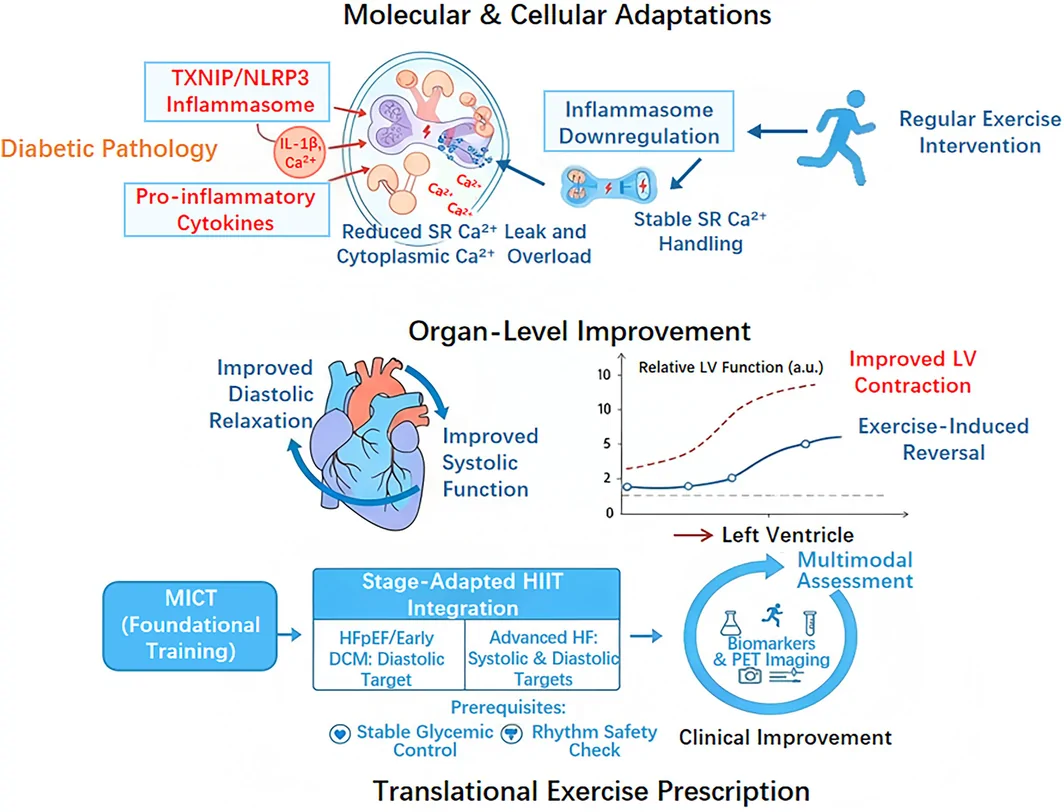
Exercise‐mediated restoration of Ca^2+^ handling and left ventricular function: From the thioredoxin‐interacting protein (TXNIP)/NLRP3 inflammasome to stage‐adapted exercise prescription. Exercise may improve Ca^2+^ handling and left ventricular function in DCM by attenuating TXNIP/NLRP3 inflammasome‐related signaling, reducing oxidative‐inflammatory amplification, and supporting stage‐adapted exercise prescription. a.u., arbitrary units; Ca^2+^, calcium; DCM, diabetic cardiomyopathy; HF, heart failure; HFpEF, heart failure with preserved ejection fraction; HIIT, high‐intensity interval training; IL‐1β, interleukin‐1 beta; LV, left ventricle/left ventricular; MICT, moderate‐intensity continuous training; NLRP3, NOD‐like receptor family pyrin domain‐containing 3; PET, positron emission tomography; SR, sarcoplasmic reticulum; TXNIP, thioredoxin‐interacting protein.

Inflammation‐related pathways further connect hyperglycemia/ROS to Ca^2+^ instability. Hyperglycemia and ROS can activate inflammasome‐associated signaling that exacerbates RyR2‐mediated Ca^2+^ leak and promotes maladaptive remodeling, while exercise‐associated suppression of inflammatory and pyroptotic signaling is consistent with more stable Ca^2+^ handling and with concurrent improvement in systolic and diastolic performance at the organ level [7, 14, 16].

Exercise is therefore positioned, within the evidence cited here, as an intervention that modulates Ca^2+^ cycling through coordinated effects on SERCA2a‐centered Ca^2+^ recycling, T‐tubule‐dependent microdomain coupling, ATP support for Ca^2+^ transport, and inflammasome‐associated inflammatory amplification in diabetes‐associated myocardial dysfunction (a broader umbrella encompassing early or subclinical myocardial impairment associated with diabetes), with mechanistic overlap with pathways implicated in DCM [7, 14, 16, 24, 31, 61, 62].

### 3.4 Inhibition of Myocardial Fibrosis and Cell Death

Myocardial fibrosis in DCM is attributed to convergent profibrotic signaling, including advanced glycation end products‐receptor for advanced glycation end products (AGEs‐RAGE) and TGF‐β/Smad, reinforced by inflammatory amplification. This network promotes collagen overproduction and cross‐linking, which reduces ventricular compliance and accelerates adverse structural remodeling [5, 7, 63, 64]. Evidence from preclinical and translational studies links structured exercise with attenuation of fibrotic remodeling and frames its effects using a “source‐sink” model (Figure 5) [5, 65, 66, 67, 68]. In this formulation, exercise is associated with reduced upstream profibrotic drive through suppression of ROS‐dependent NF‐κB‐TGF‐β/Smad signaling and inflammasome‐related inflammatory activity, while simultaneously aligning with increased extracellular matrix (ECM) turnover and restoration of ECM homeostasis, thereby opposing net collagen accumulation [5, 65, 66, 67, 68].

**FIGURE 5:**
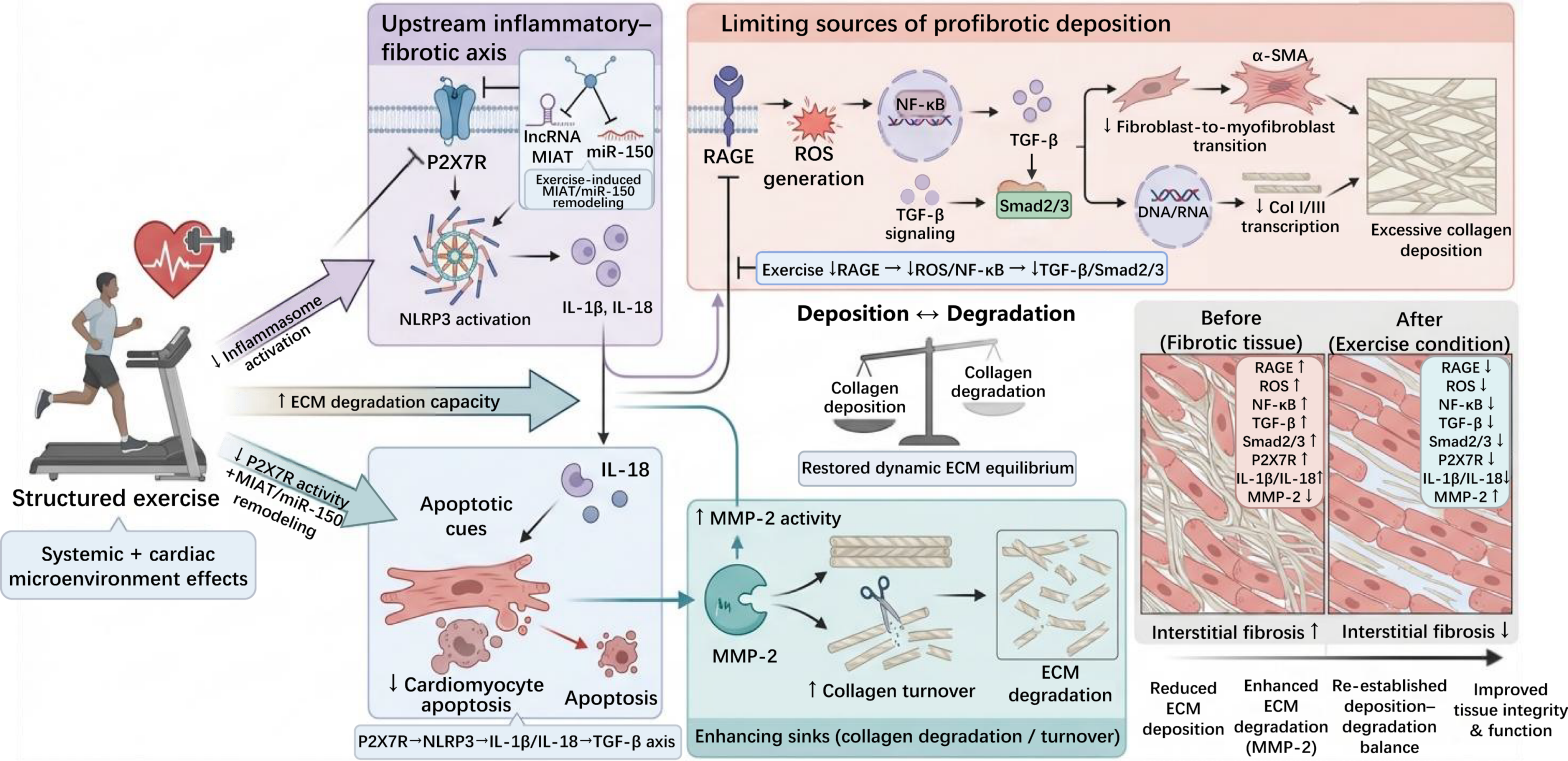
Exercise‐driven suppression of profibrotic signaling and restoration of ECM homeostasis in DCM. Exercise suppresses profibrotic and inflammatory signaling in DCM, including AGEs‐RAGE and TGF‐β/Smad‐related pathways, reduces extracellular matrix accumulation and cell death, and helps preserve myocardial structure and function. Col I/III, collagen type I/III; DNA, deoxyribonucleic acid; ECM, extracellular matrix; IL‐18, interleukin‐18; IL‐1β, interleukin‐1 beta; lncRNA, long non‐coding RNA; MIAT, myocardial infarction‐associated transcript; miR‐150, microRNA‐150; MMP‐2, matrix metalloproteinase‐2; NF‐κB, nuclear factor kappa‐B; NLRP3, NOD‐like receptor family pyrin domain‐containing 3; P2X7R, purinergic receptor P2X7; RAGE, receptor for advanced glycation end products; RNA, ribonucleic acid; ROS, reactive oxygen species; Smad2/3, SMAD family proteins 2 and 3; TGF‐β, transforming growth factor‐beta; α‐SMA, alpha‐smooth muscle actin.

Myocardial remodeling in DCM is also shaped by cardiomyocyte loss, with apoptosis and inflammasome‐mediated pyroptosis contributing to tissue injury and structural deterioration. Exercise interventions have been reported to attenuate inflammatory execution signaling and to weaken the reinforcing relationship between pyroptotic cell death and fibrotic progression, a pattern that coincides with preservation of myocardial structure and function in experimental settings [14, 69, 70, 71].

These mechanisms jointly support a model in which exercise is associated with attenuation of profibrotic signaling and inflammation‐related cardiomyocyte loss in experimental and translational diabetic settings, with relevance to DCM progression [5, 7, 63, 68].

### 3.5 miRNA and Epigenetic Regulation

Exercise training has been reported to remodel the microRNA (miRNA) profile in experimental DCM models and diabetes‐associated myocardial dysfunction, with these changes co‐occurring with indices of cardiomyocyte survival and microvascular adaptation (Figure 6) [5, 17, 18]. This coordinated pattern is consistent with a shift away from pro‐apoptotic signaling and toward improved excitation‐contraction coupling in model systems, as interpreted in the cited studies [5, 17, 18]. Mechanistic work further supports a miR‐486a‐5p‐mammalian sterile 20‐like kinase 1 (Mst1) axis: miR‐486a‐5p is reported to suppress Mst1, and the same experimental context links this suppression to enhanced mitophagy and sirtuin 3‐dependent mitochondrial quality control, accompanied by reduced cardiomyocyte apoptosis [5, 16, 17, 18, 72]. Evidence also indicates that exercise can modulate intercellular signaling across the microvasculature‐myocyte interface; specifically, training is associated with decreased cardiac microvascular endothelial cells (CMEC)‐derived exosomal Mst1 transfer to cardiomyocytes, providing a microcirculation‐related route by which endothelial changes may contribute to cardiomyocyte protection (Figure 6) [16, 18, 72].

**FIGURE 6:**
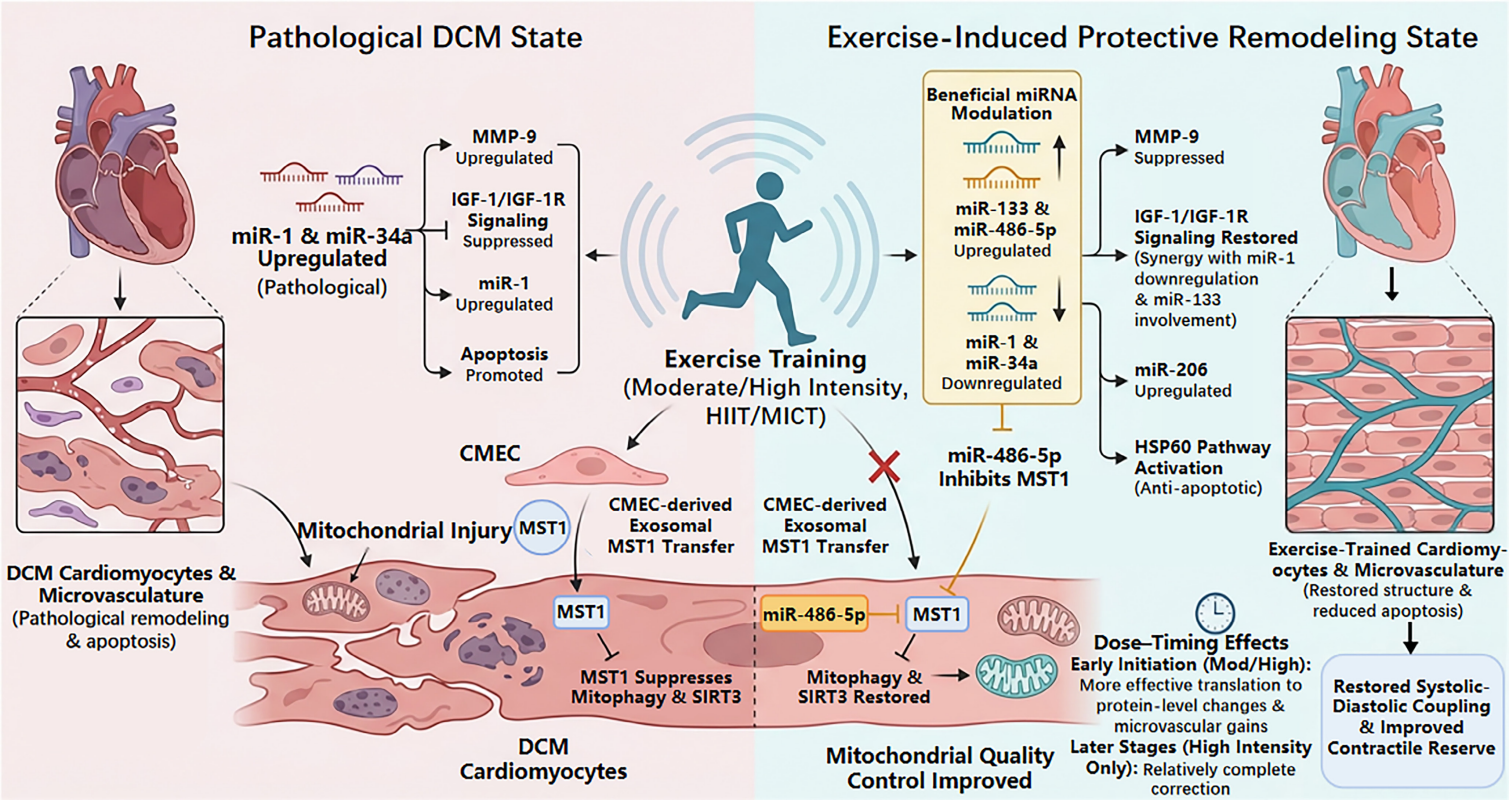
Exercise‐induced miRNA remodeling is associated with a shift toward protective remodeling in diabetic cardiomyopathy. Exercise‐induced miRNA remodeling in DCM is associated with reduced apoptosis, improved mitochondrial quality control and microvascular adaptation, and more favorable cardiac remodeling. CMEC, cardiac microvascular endothelial cell; DCM, diabetic cardiomyopathy; HIIT, high‐intensity interval training; HSP60, heat shock protein 60; IGF‐1, insulin‐like growth factor‐1; IGF‐1R, insulin‐like growth factor‐1 receptor; MICT, moderate‐intensity continuous training; miR, microRNA; miR‐1, microRNA‐1; miR‐133, microRNA‐133; miR‐206, microRNA‐206; miR‐34a, microRNA‐34a; miR‐486‐5p, microRNA‐486‐5p; MMP‐9, matrix metalloproteinase‐9; MST1, mammalian sterile 20‐like kinase 1; SIRT3, sirtuin 3.

The conversion of miRNA remodeling into downstream protein‐level changes and microvascular improvements is not uniform across disease stages and training prescriptions in preclinical evidence. Data summarized in the referenced work suggest that earlier initiation of moderate‐to‐high intensity exercise is associated with stronger alignment between miRNA shifts and protein readouts, whereas later‐stage disease models may require higher intensity stimuli to achieve changes in the same direction [17]. Circulating miRNAs have therefore been examined as candidate biomarkers of training response; in db/db models, exercise‐associated increases in plasma miR‐126 correlate with coronary microvascular density, supporting its use for longitudinal tracking of microvascular remodeling within that experimental framework [17]. Exercise‐related regulation is also described at the epigenetic level through AMPK‐sirtuin 1 (SIRT1)/sirtuin 3 (SIRT3)‐PGC‐1α signaling, which has been reported to interact with miRNA‐related regulatory modules, including the miR‐34a/SIRT1 axis, offering a plausible integrative pathway linking exercise dose with mitochondrial and vascular adaptation as argued in prior studies [5, 73].

## 4 Clinical Evidence: Efficacy and Prescription of Exercise Interventions in Diabetes‐Associated Myocardial Dysfunction

### 4.1 Clinical Effects of Different Exercise Prescriptions

Clinical evidence informing exercise prescription in DCM‐relevant metabolic cardiomyopathy phenotypes is largely derived from T2DM and subclinical cardiac abnormality cohorts [10, 11, 74, 75]. Concurrent aerobic and resistance training—typically ≥ 150 min/week of moderate‐to‐vigorous aerobic exercise (≈50%–70% maximum heart rate (HRmax)) plus 2–3 resistance sessions/week—has been associated with improved VO_2_max and insulin sensitivity and with favorable changes in LV systolic indices and vascular function (e.g., flow‐mediated dilation (FMD)) across 12‐week interventions [10, 11, 74, 75]. Imaging studies report concurrent improvements in diastolic indices and reverse‐remodeling signals; in some cohorts these changes co‐occur with reductions in hepatic fat and redistribution of peripheral adiposity, supporting co‐variation between metabolic adaptation and organ‐level function without establishing causality [11, 75]. Reports show that FMD improves and that its magnitude tracks with cardiovascular risk reduction, supporting vascular responsiveness as an intermediate endpoint [74].

Comparisons of HIIT and MICT indicate modality‐dependent differences in selected cardiac endpoints. HIIT (e.g., 3 sessions/week for 12 weeks; ~30 s at 80%–90% HRmax with ~1 min recovery) has been associated with larger improvements than MICT in several myocardial remodeling‐related measures, with generally comparable adherence [11]. Systematic reviews indicate glycemic and cardiorespiratory benefits that are at least non‐inferior to MICT, with superiority in some analyses [4]. Because HIIT imposes higher autonomic and electrophysiological demands, clinical reports support use conditional on risk stratification and monitoring; in autonomic neuropathy, overt cardiomyopathy, or elevated arrhythmic risk, initiating with MICT and transitioning to HIIT under predefined criteria is consistent with published safety considerations [76].

In high‐risk T2DM with early cardiac abnormalities but without overt heart failure, longer‐duration individualized programs (e.g., 6 months) have been associated with improved exercise tolerance, lower resting heart rate, and imaging‐reported improvements in mechanics and strain [11]. Home‐based moderate‐intensity programs have been reported to improve arterial stiffness, lipid profiles, and inflammatory markers, supporting feasibility for longitudinal management [76, 77, 78]. Across trials summarized in Table 3, when energy expenditure is approximately matched, both MICT and HIIT are commonly associated with improvements in VO_2_peak and LV structural/functional measures, serious exercise‐related adverse events are reported infrequently, and vascular endpoints can be used to test whether myocardial adaptations co‐occur with vascular improvement [11, 83].

**TABLE 3:** Clinical exercise prescriptions and imaging/functional endpoints.

Population and phenotype	Comparison	Exercise dose	Dose matching between groups	Primary imaging/functional endpoints	References
Adults with T2DM, *n* = 28, subclinical cardiac dysfunction	HIIT vs. standard care	HIIT, 3 sessions/week, 12 weeks	Unequal (exercise vs. standard care)	CMR: LV mass, LVEF, diastolic filling; CPET: VO_2_peak; liver fat	[11]
Adults with T2DM and diastolic dysfunction (pilot study)	HIIE vs. MIE within the T2DM cohort	12 weeks of endurance exercise	Different exercise intensities; not a non‐exercise control design	Speckle‐tracking echocardiography: LV twist/untwist parameters, especially timing of LV untwist rate	[79]
Adults with T2DM; HIIT *n* = 11, control *n* = 5	HIIT vs. no‐training control	HIIT for 3 months	Unequal (exercise vs. control)	Exercise echocardiography: LVEDV, LVESV, LVSV during exercise; VO_2_peak	[12]
Adults with T2DM without known cardiovascular disease; randomized *n* = 27 (HIIT *n* = 15, MIT *n* = 12), completers *n* = 19	HIIT vs. MIT	24 weeks, 3 sessions/week	Similar frequency/volume, differing intensity	CPET: VO_2_peak, Wpeak; exercise echocardiography: cardiac output, O_2_ extraction, longitudinal strain during exercise	[13]
Working‐age adults with T2DM; randomized *n* = 87, completers *n* = 76	Routine care vs. supervised aerobic exercise vs. low‐energy meal replacement	12‐week intervention	Unequal (three‐arm design)	CMR: LV peak early diastolic strain rate (primary), LV mass/volume; CPET: exercise capacity	[80]
Patients with T2DM, *n* = 80 randomized	Combined HIIT + RT vs. combined MCT + RT vs. control	1 year, 3 sessions/week	Exercise arms had similar session frequency, differing intensity	Carotid IMT, central and peripheral PWV, distensibility coefficient	[81]
Sedentary adults with early‐onset T2DM	AIT individualized by TRIMPi vs. usual physical activity recommendation/control	12 weeks	Unequal (training vs. control/usual recommendation)	Arterial stiffness, baroreflex sensitivity, blood pressure; also exercise capacity, HbA1c, body weight	[82]
Adults with T2DM (CGM‐guided exercise timing trial)	Personalized exercise timing based on CGM vs. comparator timing advice/control	Prescribed moderate‐intensity aerobic exercise timed using CGM‐derived glucose patterns	Unequal in achieved exposure/adherence	Glycemic outcomes including peak glucose; cardiometabolic/vascular outcomes including FMD, arterial stiffness, blood pressure, body composition	[19]

*Note:* Representative randomized or controlled clinical studies in adults with T2DM, with or without subclinical diabetes‐associated myocardial dysfunction. Exercise interventions included HIIT, moderate‐intensity continuous/moderate‐intensity training, aerobic interval training, resistance training, or CGM‐guided exercise timing. Outcomes covered cardiac structure/function, exercise capacity, vascular function/arterial stiffness, glycemic outcomes, and body composition.

Abbreviations: BRS, baroreflex sensitivity; CGM, continuous glucose monitoring; CMR, cardiac magnetic resonance; CO, cardiac output; CPET, cardiopulmonary exercise testing; FMD, flow‐mediated dilation; HbA1c, glycated hemoglobin; HIIE, high‐intensity interval exercise; HIIT, high‐intensity interval training; IMT, intima‐media thickness; LV, left ventricle/left ventricular; LVEDV, left ventricular end‐diastolic volume; LVEF, left ventricular ejection fraction; LVESV, left ventricular end‐systolic volume; LVSV, left ventricular stroke volume; MCT, moderate continuous training; MIE, moderate‐intensity exercise; MIT, moderate‐intensity training; PWV, pulse wave velocity; RT, resistance training; T2DM, Type 2 diabetes mellitus; TRIMPi, training impulse index; VO_2_peak, peak oxygen uptake; Wpeak, peak work rate.

### 4.2 Safety and Risk Control of Exercise Prescriptions

Exercise prescription in diabetic cardiomyopathy should be preceded by risk stratification to define an individualized safety boundary and identify conditions requiring modification or temporary deferral. Assessment should integrate disease course, prior cardiovascular events, and cardiac/renal complications. Resting electrocardiography and echocardiography should document left ventricular ejection fraction (LVEF), diastolic function, and myocardial strain; when clinical uncertainty persists, NT‐proBNP and CPET can be used to evaluate silent ischemia, characterize ventilatory/circulatory limitations, and set intensity thresholds. Diabetes‐related comorbidities that increase exercise risk (neuropathy, nephropathy, proteinuria, proliferative retinopathy, and foot complications) should be assessed concurrently, with footwear and foot‐care measures implemented to reduce preventable adverse events [1, 20, 84, 85, 86].

Workloads should be progressed within predefined target heart‐rate zones or threshold power ranges with real‐time heart rate and blood pressure monitoring. Glucose monitoring should be intensified during early training phases or intensive adjustment of glucose‐lowering therapy, with carbohydrate planning around sessions; exercise should be suspended when blood glucose is < 3.9 mmol/L or prodromal hypoglycemic symptoms occur. Because glucose‐lowering regimens differentially determine hypoglycemia risk and may influence session‐day hemodynamic tolerance (e.g., insulin/sulfonylureas vs. sodium‐glucose cotransporter 2 (SGLT2) inhibitors/glucagon‐like peptide‐1 (GLP‐1) receptor agonists), protocols should prespecify medication context and individualized glucose‐management procedures during training sessions. Chest pain, progressive dyspnea, presyncope, sustained arrhythmias, or marked blood pressure fluctuations should prompt immediate cessation and medical evaluation.

Progression should align with baseline activity level and phenotype‐specific risk: insufficiently active, high‐risk individuals can start with moderate‐intensity continuous training, integrating interval and resistance components as tolerated. In remote or home‐based programs, mobile health technologies and structured follow‐up can document adherence, capture safety‐relevant signals, and support timely adjustment while maintaining safety standards [1, 20, 84, 85, 86, 87, 88, 89, 90].

## 5 Biomarkers and Monitoring for Exercise Interventions in Diabetes‐Associated Myocardial Dysfunction

### 5.1 Framework and Stratified Applications of Potential Biomarkers

Exercise monitoring in diabetes‐associated myocardial dysfunction can be organized across molecular, organ, and whole‐body levels to guide iterative prescription. BNP/NT‐proBNP reflects wall stress and filling pressure but should be interpreted with baseline risk and training‐related variation; paired GLS provides functional corroboration [7, 21]. H‐FABP may detect short‐term load‐related myocardial injury, particularly in patients with coronary or ischemic risk [21]. IGFBP‐7 is associated with diastolic dysfunction and extracellular‐matrix remodeling, whereas sustained CT‐1 elevation may indicate maladaptive remodeling [7]. Circulating miRNA patterns (e.g., ↑miR‐126/miR‐133/miR‐486 and ↓miR‐15a/b, miR‐499, miR‐34a) remain exploratory markers of mechanism and response heterogeneity and should be interpreted with clinical and functional measures [17, 91, 92, 93, 94, 95]. Integrating biomarkers with imaging and exercise capacity may link risk, biological response, and prescription refinement across disease stages [7, 17, 21, 91] (Table 4).

**TABLE 4:** Overview of candidate biomarkers.

Domain	Marker(s)	DCM‐specific evidence focus	Use in exercise monitoring	References
Wall stress/risk	NT‐proBNP (±BNP)	In patients with subclinical diabetic cardiomyopathy, higher NT‐proBNP has been associated with worse health status and reduced functional capacity, but not with echocardiographic abnormalities.	Baseline risk/functional stratification and longitudinal trend monitoring; interpret alongside echocardiography and exercise‐capacity measures.	[20]
Clinical DCM phenotype/phenotype enrichment	BNP (context: troponin, galectin‐3)	In symptomatic patients with T2DM and preserved LVEF, BNP was higher in DCM‐HFpEF and, together with TRVmax and CAVI, independently predicted DCM, whereas galectin‐3 did not significantly differ between groups.	Baseline phenotype enrichment/risk screening; not suitable as a standalone exercise‐response marker; interpret together with echocardiographic and arterial‐stiffness measures.	[96]
Myocardial injury (candidate)	H‐FABP	Listed as candidate early injury marker in DCM biomarker reviews.	Exploratory “dose‐safety” alert; not standalone.	[97]
Fibrosis/diastolic remodeling (emerging)	IGFBP‐7	Included in recent DCM biomarker frameworks; evidence for phenotype enrichment or early diagnostic discrimination remains preliminary.	Exploratory phenotype enrichment/research tracking; not suitable as a standalone monitoring marker.	[98]
Microvascular adaptation (exercise)	miR‐126	Exercise upregulated cardiac miR‐126/angiogenic signaling in diabetic rats.	Research response readout; pair with perfusion/endothelial indices.	[99]
miRNA‐regulated protection in diabetes	miRNA network (incl. miR‐126)	Exercise preserves coronary/cardiac function via miRNA regulation in diabetic models.	Mechanistic support for response heterogeneity (research).	[17]
Apoptosis/contractile remodeling	miR‐133a	Exercise increases miR‐133a and improves injury/apoptosis balance in diabetic rats.	Mechanistic readout (research/translation).	[100]
Anti‐apoptotic pathway marker	miR‐486a‐5p‐Mst1 axis	Exercise protection in DCM linked to miR‐486a‐5p‐Mst1 inhibition.	Pathway‐level validation (research).	[18]
Fibrosis response (exercise)	miR‐34a	Swimming reduced fibrosis via miR‐34a‐related signaling in T2DM rats.	Fibrosis‐response marker for modality comparisons (preclinical).	[37]

*Note:* miRNAs, IGFBP‐7, and H‐FABP should be regarded as investigational or phenotype‐enrichment markers in DCM. Routine monitoring still relies mainly on symptoms, imaging, exercise capacity, and validated cardiac biomarkers.

Abbreviations: BNP, B‐type natriuretic peptide; CAVI, cardio‐ankle vascular index; DCM, diabetic cardiomyopathy; H‐FABP, heart‐type fatty acid‐binding protein; HFpEF, heart failure with preserved ejection fraction; IGFBP‐7, insulin‐like growth factor‐binding protein 7; LVEF, left ventricular ejection fraction; NT‐proBNP, N‐terminal pro‐B‐type natriuretic peptide; TRVmax, maximal tricuspid regurgitation velocity.

### 5.2 Longitudinal Monitoring Framework and Evaluation Rhythm

Baseline assessment provides a reproducible reference for longitudinal monitoring across structural, functional, and metabolic domains, enabling stratified exercise prescription and subsequent adjustment on comparable measurements [7, 21]. Echocardiography defines the structural‐functional domain using left ventricular ejection fraction (LVEF), global longitudinal strain (GLS), interventricular septal/posterior wall thickness, and the left atrial volume index; three‐dimensional echocardiography and speckle‐tracking techniques, when available, increase sensitivity to mild fibrosis‐related remodeling and subtle extracellular volume changes [7, 21]. Functional capacity and intensity stratification are assessed by CPET through VO_2_max, peak workload, and ventilatory response, with the oxygen pulse‐heart rate reserve relationship informing individualized intensity targets [21]. The metabolic‐vascular context is characterized by brachial artery flow‐mediated dilation (FMD) combined with fasting glucose/insulin and the homeostasis model assessment of insulin resistance (HOMA‐IR). Integrated interpretation across these domains reduces bias associated with reliance on a single parameter [7, 21].

### 5.3 Process Monitoring and Prescription Iteration

Process monitoring can follow a tiered schedule matched to response kinetics, so prescription iteration is based on trajectories. Adherence‐related indices (fasting glucose, body weight/waist circumference, resting heart rate, and home‐based heart rate variability (HRV)) can be re‐checked every 4–8 weeks to verify implementation fidelity and early metabolic/autonomic shifts. Echocardiographic strain and diastolic parameters can be reassessed every 12 weeks and interpreted with quantitative NT‐proBNP/BNP (or H‐FABP in high‐risk individuals) to evaluate structural‐functional response while monitoring loading and injury signals [7, 17, 21].

Circulating miRNAs can be sampled every 12–16 weeks, focusing on upward trends in miR‐126, miR‐133a, and miR‐486a‐5p and downward trends in miR‐1, miR‐34a, and miR‐15a/b. Concordance between miRNA trajectories and GLS/BNP improvement may support mechanistic attribution in research settings (“molecular‐organ dual concordance” as an exploratory construct), whereas discordance should primarily prompt reassessment of adherence, measurement timing, and training dose implementation; any linkage to inflammatory/pyroptotic activation should be framed as hypothesis‐generating rather than actionable for clinical decision‐making [17, 91, 92, 93, 95, 101].

For a prominent metabolic syndrome phenotype, fasting insulin, glycated hemoglobin, and FMD can be monitored to assess whether peripheral vascular responsiveness is limiting gains and to guide adjustment toward vascular‐metabolic targets [7, 17, 21].

### 5.4 Endpoint Evaluation and Clinical Translation

A comprehensive “end‐of‐cycle” evaluation at 6 months can be used as a standardized time point for assessing response and informing subsequent exercise prescription in diabetes‐associated myocardial dysfunction. The assessment integrates imaging indices (GLS, LVEF, diastolic function), functional indices (VO_2_max, anaerobic threshold (AT), 6‐min walk distance (6MWD)), vascular indices (e.g., FMD), and established biomarkers (NT‐proBNP/BNP), with optional investigational measures (e.g., H‐FABP, fibrosis‐related markers, and circulating miRNAs) for mechanistic attribution in research settings. Considered jointly, these measures may support an operational categorization of response patterns (e.g., consolidation/maintenance vs. re‐stratification of training dose), pending prospective validation in etiologically adjudicated DCM cohorts [7, 17, 21, 92].

This composite pattern is proposed for research interpretation, not as a diagnostic or clinical threshold. A favorable trajectory may combine an approximately ≥ 30% NT‐proBNP reduction, ≈2‐percentage‐point GLS improvement, and increased VO_2_max; miRNA components remain exploratory. Prospective validation in adjudicated DCM cohorts is required [7, 17, 21, 92]. A BNP/NT‐proBNP decline without FMD improvement may indicate incomplete myocardial‐vascular coupling and support greater emphasis on combined aerobic‐resistance training and recovery. Discordant miRNA and imaging or histological responses should prompt reassessment of adherence, measurement timing, and training dose; links to inflammatory or pyroptotic activation remain hypothesis‐generating [17, 69, 70, 102].

This monitoring scheme is anchored to the triad of “biomarkers‐imaging‐exercise capacity” and offers a structured framework for iterative dose adjustment and re‐evaluation in diabetes‐associated myocardial dysfunction and DCM‐relevant phenotypes, pending validation of clinically interpretable thresholds and implementation feasibility [7, 17, 21, 92].

## 6 Research Gaps and Clinical Translation Perspectives

Despite consistent evidence that exercise improves left ventricular structure and functional capacity in diabetes‐associated myocardial dysfunction and DCM‐relevant phenotypes (predominantly derived from T2DM cohorts with subclinical myocardial abnormalities), reproducible translation is limited by heterogeneous trial design and implementation. Broad eligibility criteria are often not paired with prespecified stratification by sex, age, or metabolic phenotype (e.g., obese vs. lean T2DM), and reporting of randomization, blinding, and adherence is frequently incomplete. Across major trials (e.g., HF‐ACTION, HEART Camp, REHAB‐HF), efficacy interpretation varies with adherence ascertainment, especially when device‐derived objective data are combined with self‐report; the absence of a unified definition and metric limits cross‐trial comparability and external validity [103, 104, 105, 106, 107, 108].

Mechanistic evidence implicates mitochondrial biogenesis, redox balance, Ca^2+^ handling, and inflammation‐fibrosis pathways, yet most trials lack prespecified molecular or imaging endpoints aligned to these mechanisms, leaving mechanistic attribution uncertain [22]. Exercise‐associated miRNA remodeling remains insufficiently validated in clinically adjudicated DCM cohorts and may be stage‐dependent [91, 93, 95, 109]. Protein biomarkers (H‐FABP, CT‐1, IGFBP‐7) have been explored, but clinically actionable cut‐offs, minimal detectable change (MDC), and minimal clinically important difference (MCID) are not established, and modifiers (sex, age, pharmacotherapy, adherence) remain poorly quantified [4, 21].

Future multicenter randomized controlled trials (RCTs) should prespecify composite endpoints integrating strain‐based imaging, VO_2_peak/6‐min walk test (6MWT), and clinical events; define adherence a priori as a co‐primary endpoint using unified objective criteria; and apply phenotype‐ and risk‐adapted prescriptions with staged escalation and safety monitoring for arrhythmia‐prone or high‐scar phenotypes [102, 103, 104, 105, 106, 107, 108, 110, 111, 112, 113]. Standardization across prescription, implementation, and mechanistic measurements, together with cross‐species dose/intensity mapping, is required to improve reproducibility and scalability [22, 91, 93, 104, 114, 115].

## 7 Conclusion

Regular, monitorable exercise is associated with improved diabetes‐related myocardial dysfunction through pathways relevant to DCM, although direct evidence in adjudicated DCM cohorts remains limited. The proposed workflow therefore applies mainly to T2DM‐associated early‐to‐intermediate phenotypes, and advanced DCM requires prospective validation. Benefits span metabolic remodeling, ventricular structure and function, exercise tolerance, and metabolic phenotype when dose and adherence are sustained and documented. For clinically stable patients, a pragmatic regimen is ≥ 150 min/week of moderate‐intensity aerobic exercise (≈50%–70% HRmax) plus 2–3 resistance sessions/week; selected lower‐risk individuals may add HIIT at ≈80%–90% HRmax after risk stratification and with longitudinal monitoring. Guideline‐supportive evidence requires harmonized dose and adherence reporting, validated response and safety thresholds for BNP/NT‐proBNP, defined roles for investigational markers (H‐FABP, IGFBP‐7, circulating miRNAs), and validated mappings between animal workloads and human METs/%HRR.

## Author Contributions

**Xiaoya Li:** conceptualization, literature search, writing – original draft, visualization. **Zhiqiang Wang:** conceptualization, literature search, writing – original draft, writing – review and editing. **Jingjing Liu:** supervision, validation, writing – review and editing.

## Funding

The authors have nothing to report.

## Ethics Statement

Ethics approval and informed consent were not required because this article is a narrative review based exclusively on previously published literature and did not involve human participants, identifiable human data, or animal experiments conducted by the authors.

## Consent

Consent for publication was not required because this article contains no identifiable personal information, images, videos, recordings, or individual case details.

## Conflicts of Interest

The authors declare no conflicts of interest.

## Data Availability

Data sharing is not applicable to this article because no datasets were generated or analyzed during the preparation of this review. All information discussed in this article was obtained from previously published studies cited in the manuscript.
